# Delayed Meal Timing, a Breakfast Skipping Model, Increased Hepatic Lipid Accumulation and Adipose Tissue Weight by Disintegrating Circadian Oscillation in Rats Fed a High-Cholesterol Diet

**DOI:** 10.3389/fnut.2021.681436

**Published:** 2021-07-01

**Authors:** Daeun Kim, Fumiaki Hanzawa, Shumin Sun, Thomas Laurent, Saiko Ikeda, Miki Umeki, Satoshi Mochizuki, Hiroaki Oda

**Affiliations:** ^1^Laboratory of Nutritional Biochemistry, Department of Applied Molecular Biosciences, Nagoya University, Nagoya, Japan; ^2^Department of Nutritional Sciences, Nagoya University of Arts and Sciences, Nissin, Japan; ^3^Institute of Innovation for Future Society, Nagoya University, Nagoya, Japan; ^4^Faculty of Food Science and Nutrition, Beppu University, Beppu, Japan; ^5^Faculty of Education, Oita University, Oita, Japan

**Keywords:** circadian rhythm, breakfast skipping, high-cholesterol diet, fatty liver, body temperature, delayed meal timing

## Abstract

**Background:** To investigate whether shifted timing of eating, breakfast skipping, induces alterations in the circadian clock and abnormal lipid metabolism, we have established a delayed meal timing (DMT) protocol for rats, which started eating food 4 h delay. In the present study, control and DMT rats were fed a high-cholesterol diet during zeitgeber time (ZT) 12-24 and ZT 16-4, respectively. The DMT protocol increased the hepatic lipids and epididymal adipose tissue weight without changes in food intake and body weight. The surge in body temperature was delayed by 4 h in the DMT group, suggesting that energy expenditure was decreased in response to DMT. The peaks of the diurnal rhythm of serum non-esterified fatty acids and insulin were delayed by 2 and 4 h due to DMT, respectively. The oscillation peaks of hepatic *de novo* fatty acid synthesis gene expression was delayed by 4 h in response to DMT, whereas the peak of hepatic clock genes were 2 h delayed or not by DMT. Although metabolic oscillation is considered to be controlled by clock genes, the disintegration rhythms between the clock genes and lipid metabolism-related genes were not observed in rats fed a high-fat diet in our previous study. These data suggest that the circadian rhythm of *de novo* fatty acid metabolism is regulated by timing of eating, but is not directly controlled by clock genes. The present study suggests that breakfast skipping would complicate fatty liver and body fat accumulation.

## Introduction

Daily rhythms in physiology and behavior, including hormone secretion, sleep-wake cycle, and body temperature, are controlled by circadian clock in mammals. The circadian clock in mammals is organized hierarchically, in a multiple oscillator system (1). The master clock, located in the hypothalamic suprachiasmatic nuclei (SCN) in the brain (2), orchestrates peripheral oscillators in extra-SCN tissues, such as the liver and other organs. The heterodimers of CLOCK (Table 1) and BMAL1 activate the transcription of the *PER*s and *CRY*s genes. PER and CRY form heterodimers and suppress the transactivation of CLOCK/BMAL1 dimers, which form a critical negative feedback loop (3). The negative feedback loop involves the other clock genes, such as, *DEC, REV-ERB*s, and *ROR*α. Some clock genes, such as *REV-ERB*s (4, 5), *ROR*α (6), *E4BP4* (7), *DBP, HLF*, and *TEF* (8), directly regulate genes associated with metabolism. In particular, *DBP* is a liver-enriched transcription factor that controls bile acid metabolism in the liver through the regulation of CYP7A1, which is the most important enzyme in the rate-limiting step of bile acid synthesis (9). The list of gene names we used in the present study was provided in Table 1.

**Table 1:** List of gene names used in the present study.

**Gene names**	**Accession No**.	**Translated products**
*ACACA*	NM_022193.1	Acetyl-CoA carboxylase alpha
*ACLY*	NM_016987.2	ATP citrate lyase
*ACOX1*	NM_017340.2	Acyl-CoA oxidase 1
*APO E*	NM_001270681.1	Apolipoprotein E
*BMAL1*	NM_024362.2	Brain and muscle Arnt-like protein 1
*CLOCK*	NM_021856.2	Circadian locomoter output cycles protein kaput
*CPT1α*	NM_031559.2	Carnitine palmitoyltransferase 1A
*CRY1*	NM_198750.2	Cryptochrome 1
*CRY2*	NM_133405.2	Cryptochrome 2
*CYP7A1*	NM_012942.2	Cytochrome P450 family 7 subfamily A member 1
*CYP8B1*	NM_031241.1	Cytochrome P450 family 8 subfamily B member 1
*DBP*	NM_012543.3	D site of albumin promoter binding protein
*DEC1*	NM_053328.1	Differentiated embryo chondrocyte 1
*DEC2*	XM_002729454.6	Differentiated embryo chondrocyte 2
*E4BP4*	NM_053727.2	Nuclear factor, interleukin 3 regulated
*ELOVL6*	NM_134383.2	Elongation of long-chain fatty acids family member 6
*FAS*	NM_017332.1	Fatty acid synthase
*FGF21*	NM_130752.1	Fibroblast growth factor 21
*FXR*	NM_021745.1	Nuclear receptor subfamily 1, group H, member 4
*HLF*	NM_001100764.1	Hepatic leukemia factor
*HMG-CoAR*	NM_013134.2	3-hydroxy-3-methylglutaryl-CoA reductase
*HMG-CoAS*	NM_017268.1	3-hydroxy-3-methylglutaryl-CoA synthase 1
*PER1*	NM_001034125.1	Period 1
*PER2*	NM_031678.1	Period 2
*PPARα*	NM_013196.1	Peroxisome proliferator activated receptor alpha
*REV-ERBα*	NM_001113422.1	Nuclear receptor subfamily 1, group D, member 1
*REV-ERBβ*	NM_147210.2	Nuclear receptor subfamily 1, group D, member 2
*RORα*	XM_008766408.2	Nuclear receptor subfamily 1, group F, member 1
*SHP*	NM_057133.1	Small heterodimer partner
*SREBP1*	NM_001276708.1	Sterol regulatory element binding protein 1c
*SREBP2*	NM_001033694.1	Sterol regulatory element binding protein 2
*TEF*	NM_019194.2	Thyrotrophic embryonic factor

Feeding behavior and the circadian clock are closely linked to human health. Our previous study indicated that irregular feeding caused hypercholesterolemia and disrupted the hepatic circadian rhythms of clock genes (10). Furthermore, the circadian rhythm of the *CYP7A1* gene in nocturnal expression is advanced by irregular feeding behavior (10). We reported that insulin acts as a major synchronizer of the hepatic clock (11). Insulin regulates post-translational modification of BMAL1 protein and affects the hepatic clock (12). Reportedly, time-restricted feeding provided protection against abnormal lipid metabolism in mice fed a high-fat diet (13). Moreover, time-restricted feeding within the active phase ameliorated fatty liver in rats fed a high-sucrose diet (14). These studies indicate that feeding time is an important factor for in the circadian rhythm of clock genes and lipid metabolism.

In modern society, several individuals have irregular work schedules and occasionally develop disorders associated with irregular rhythms. Reportedly, shift-workers such as nurses and flight attendants have a higher risk of cancer and cardiovascular diseases (15–18). Breakfast skipping is a major irregular feeding behavior in the modern society. Almost 10–30% of American and European children and adolescents regularly skip breakfast (19–21). Approximately 28% individuals in the age group of 20–29 years skip breakfast in Japan (22). Several epidemiological studies have reported that breakfast skipping is a risk factor associated with health problems such as metabolic syndrome (23, 24), type 2 diabetes (25), and coronary heart disease (26). In addition, some studies have suggested that breakfast skipping is related to learning performance in children and adolescents (20, 27, 28). Therefore, breakfast is considered the most important meal of the day for both children and adults (23–28). However, the molecular mechanism underlying the abnormal metabolic conditions induced by breakfast skipping remains unknown.

We hypothesized that long-term breakfast skipping induces metabolic disorders owing to abnormalities in the circadian clock. We recently developed a delayed meal timing (DMT) protocol for rats, which is a one kind model of breakfast skipping and investigated whether it affected lipid metabolism by altering the circadian rhythm in the liver (29). DMT promoted body weight gain in rats fed a high-fat diet without changing the food intake. Moreover, DMT induced a delay in circadian oscillations for clock genes as well as lipid metabolism-related genes in the liver. And insulin was also 4 h delayed by DMT.

Feeding a high-cholesterol diet induced fatty liver as well as hypercholesterolemia (30), whereas a high-fat diet induced obesity and insulin resistance (31). High-cholesterol diet for rats is an experimental model for fatty liver and hypercholesterolemia, as a high-cholesterol diet indues massive lipid accumulation in the rat liver. Fatty liver is thought to be a risk factor for non-alcoholic fatty liver disease (NAFLD) in humans (32). In the present study, we hypothesized that DMT with a high-cholesterol diet promotes fatty liver and hypercholesterolemia, and induces abnormalities in the circadian clock genes rhythms. We found that DMT induced abnormalities in lipid metabolism, such as increase in adipose tissue weight and hepatic lipids without changing the hypercholesterolemia status. The circadian rhythms of hepatic lipid metabolism-related genes were delayed by ~2–4 h, although the oscillations of hepatic clock gene expression were relatively consistent. The disintegration of the hierarchical circadian system in DMT with a high-cholesterol diet suggested that the circadian oscillation of hepatic lipid metabolism was controlled by the feeding time rather than by clock genes under short-term DMT.

## Materials and Methods

### Animals

The animal study was approved by the Animal Care Committee of Nagoya University (Approval No. 2014110401) and conducted in compliance with the Rules and Regulations of the Guide for the Care and Use of Laboratory Animals, Nagoya University. All surgical procedures were performed under isoflurane anesthetization, and all efforts were made to minimize suffering. Fifty-five 5-week-old male Wistar rats were purchased from Japan SLC (Shizuoka, Japan). The rats were housed in individual wire-bottomed cages and maintained under a 12 h light cycle [zeitgeber time (ZT) 0-ZT 12]. The rats were fed a commercial stock diet (Lab MR Breeder, Nosan Co., Yokohama, Japan) for 3 days, followed by a basal diet for 10 days. The composition of the basal diet is presented in Table 2. Nine days after the rats were obtained, temperature data loggers (KN laboratories Inc., Osaka, Japan) were implanted into their intraperitoneal cavity. The rats were allowed to recover for 4 days after the surgery. Thirteen days after the rats were obtained, they were divided into two groups (*n* = 27 in the control group, *n* = 28 in the DMT group) by matching body weight. The rats were then fed a high-cholesterol diet for 16–17 days during the experimental period. As rats are nocturnal animals, they consume ~80% of their daily food intake during ZT 12-24 (33). The rats in the control group (*n* = 27) and DMT groups (*n* = 28) were provided access to a high-cholesterol diet from ZT 12 to ZT 24 and ZT 16 to ZT 4, respectively. In the current study, the rats were provided a sufficient portion of the diet at once before the feeding time and free access to the diet was provided during the feeding period (12 h). The control group was fed a diet on ZT 12, and the DMT group was fed a diet on ZT 16. In the DMT protocol, we fixed the feeding duration at 12 h in both groups, because it has been reported that duration of the feeding affects lipid metabolism (13). The 8-h feeding schedule dramatically improved the high-fat diet-induced abnormal lipid metabolism (13). We applied the DMT protocol as a one kind of breakfast skipping model, although the feeding time was delayed by 4 h in the DMT protocol. Moreover, individuals who skipped breakfast are related to late dinner at night. This habit was also associated with metabolic syndrome in Japanese population (34). Water was provided *ad libitum* throughout the experimental period. The composition of a high-cholesterol diet is presented in Table 2. We added 0.25% sodium cholate to the diet (Table 2), as sodium cholate drastically increased the cholesterol absorption to increase serum total cholesterol levels in rats (35). A high-cholesterol diet with cholate induces fatty liver and hypercholesterolemia. The food was removed at ZT 24 in the control group and at ZT 4 in the DMT group. After the food was removed, the body weight, and total daily food intake were calculated. To analyze the pattern of their food intake during the feeding period, we measured the amount of their food intake three times at 4 h intervals. In order to avoid influencing the food intake, the food was carefully removed from each cage, measured, and immediately returned. Three or four rats from each group were sacrificed at an interval of 4 h from ZT 2 on day 16 to next ZT 2 on day 17. The rats were sacrificed by decapitation without anesthetization to minimize the influence of stress on the serum corticosterone levels. The livers were harvested and frozen immediately using liquid nitrogen. The frozen samples were stored at −80°C.

**Table 2:** Composition of a basal diet and a high-cholesterol diet.

**Ingredient**	**Basal**	**High-cholesterol**
	**g/kg diet**	**g/kg diet**
Casein^a^	200	200
Vitamin mixture^b^	10	10
Mineral mixture^c^	35	35
Choline chloride	2	2
Corn oil	50	50
Cellulose	50	50
Starch	435	427
Sucrose	218	213.5
Cholesterol	-	10
Sodium cholate	-	2.5

a *Crude protein : 84.3%*.

b *AIN93-VX*.

c *AIN93-MX*.

### Measurement of Spontaneous Activity

The spontaneous activities of the control and DMT rats (*n* = 3) were monitored in separated individual transparent-plastic cages using a LOCOMO LS-8 system (Melquest, Toyama, Japan) for 1 day. The transparent-plastic cage was placed inside the sensor panel (28.0 × 44.0 cm), and the photobeam interruptions were counted by the interface when the rat moved in the cage. The activities were counted every 10 min for 24 h. The rats were allowed to acclimatize to the measuring environment for 1 h before the actual measurement.

### Measurement of Body Temperature

The body temperature of the control (*n* = 6) and DMT rats (*n* = 7) was measured using a temperature data logger, as described above. Body temperature was recorded every 10 min continuously during the experimental period with a resolution of 0.1°C. The collected data were analyzed at an interval of 60 min using the Rh Manager program (KN laboratories Inc., Osaka, Japan).

### Biochemical Analysis

Liver lipids were extracted by using the method described by Folch et al. (36). Total liver lipids were measured gravimetrically. Hepatic cholesterol, triglycerides, and phospholipids in the lipid extract were measured enzymatically (T-CHO and TG-EN; Kainos Laboratories, Tokyo, Japan, Phospholipids C-Test, Wako Pure Chemical Industries, Osaka, Japan). Serum glucose, total cholesterol, triglyceride, non-esterified fatty acids (NEFA), and bile acids were measured enzymatically by using commercial kits (Glucose CII-test, Triglyceride E-test, Cholesterol C-test, NEFA C-test, TBA-test; Wako Pure Chemical Industries, Osaka, Japan). The insulin and corticosterone levels were measured using rat-specific enzyme immunoassay kits [Rat insulin ELISA kit (#M1101); Morinaga Institute of Biological Science, Yokohama, Japan, Corticosterone ELISA kit (#EC3001-1); Assaypro, MO, USA].

### Total RNA Extraction and Real-Time Quantitative Polymerase Chain Reaction (PCR)

Total RNA was extracted from the liver and epididymal adipose tissue using the method described by Chomczynski and Sacchi (37). After the RNA quality was assessed by using northern blotting, cDNA was synthesized using a High Capacity cDNA Reverse Transcription Kit (Thermo Fisher Scientific). Real-time quantitative PCR was performed using the Power SYBR™ Green PCR Master Mix (Thermo Fisher Scientific) and analyzed using the StepOnePlus Real-Time PCR System (Thermo Fisher Scientific). The primer sequences are provided in Supplementary Table 1. The mRNA levels were normalized relative to the mRNA levels of apolipoprotein E (Apo E) in the liver and 18S RNA in the epididymal adipose tissue.

### Rhythmicity Analysis

The oscillations in body temperature, serum lipids, hormones, and gene expression of hepatic clock and lipid metabolism were analyzed by using JTK_CYCLE implemented on R (38). The peak time and amplitude of the rhythms were analyzed using JTK_CYCLE for a period of 24 h. Rhythmicity was confirmed at *p* < 0.05.

### Statistics Analysis

**Figure 1:**
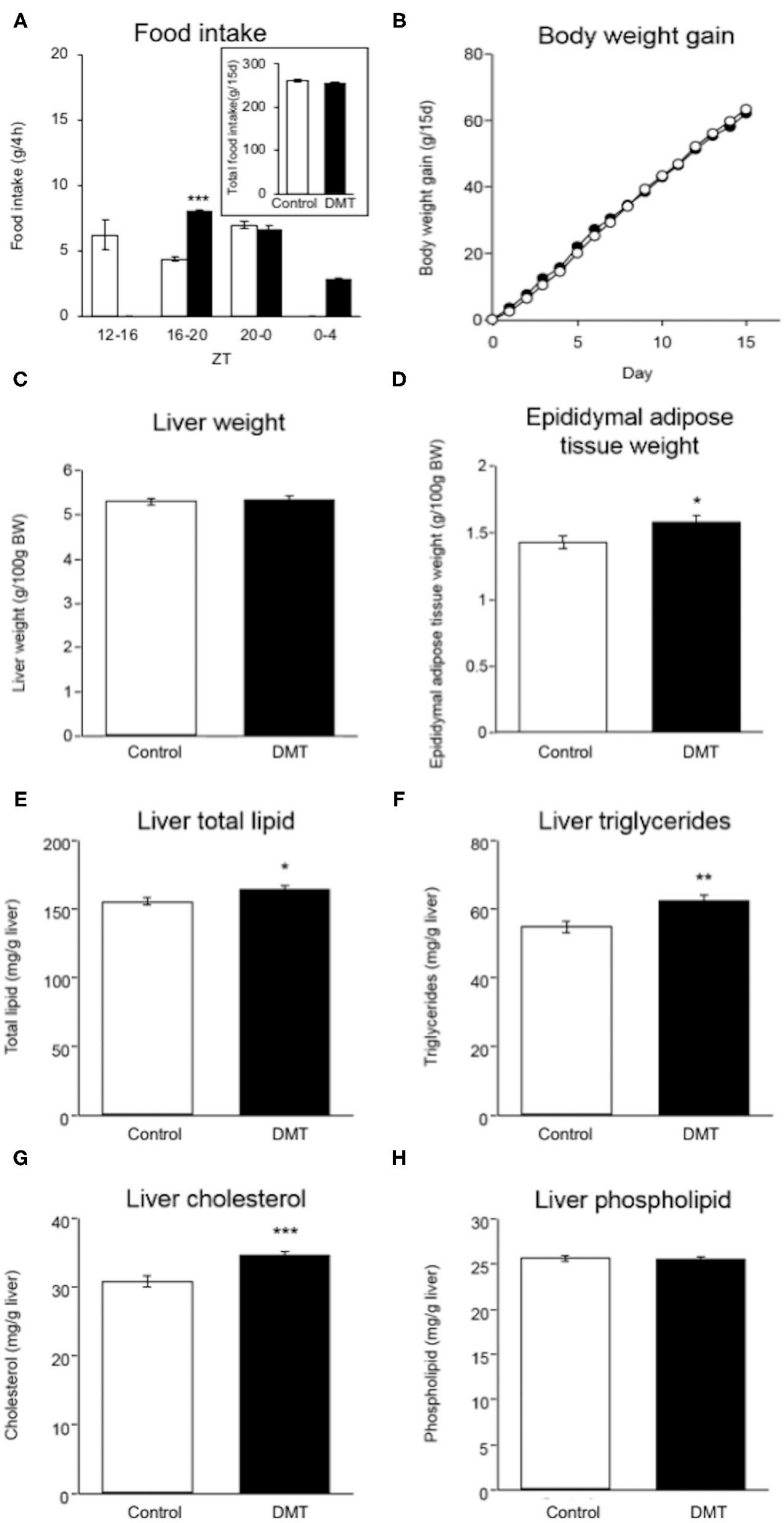
DMT induced increased adipose tissue weight and hepatic lipids. **(A)** Food intake was measured at 4 h intervals on day 14–15. (**A**: inset) Total food intake in both groups during the experimental period. **(B)** Body weight gain in both groups were measured during ZT 4 every day for 15 days. The open circles indicate the control group (°) and the solid circles indicate the DMT group (•). **(C)** Liver weight and **(D)** epididymal adipose tissue weight were measured when the organs were harvested. The livers and epididymal adipose tissues were harvested at seven time points with 4 h interval on day 16–17. The amount of liver lipids including **(E)** total lipid, **(F)** triglycerides, **(G)** cholesterol, and **(H)** phospholipids was measured. Data are presented as mean ± SEM (*n* = 27 in the control group, *n* = 28 in the DMT group). Statistical analysis was performed using the Student's *t*-test. The *, **, and *** indicate significant difference (*p* < 0.05, *p* < 0.01, and *p* < 0.001) compared to control group, as measured using the Student's *t*-test.

**Figure 2:**
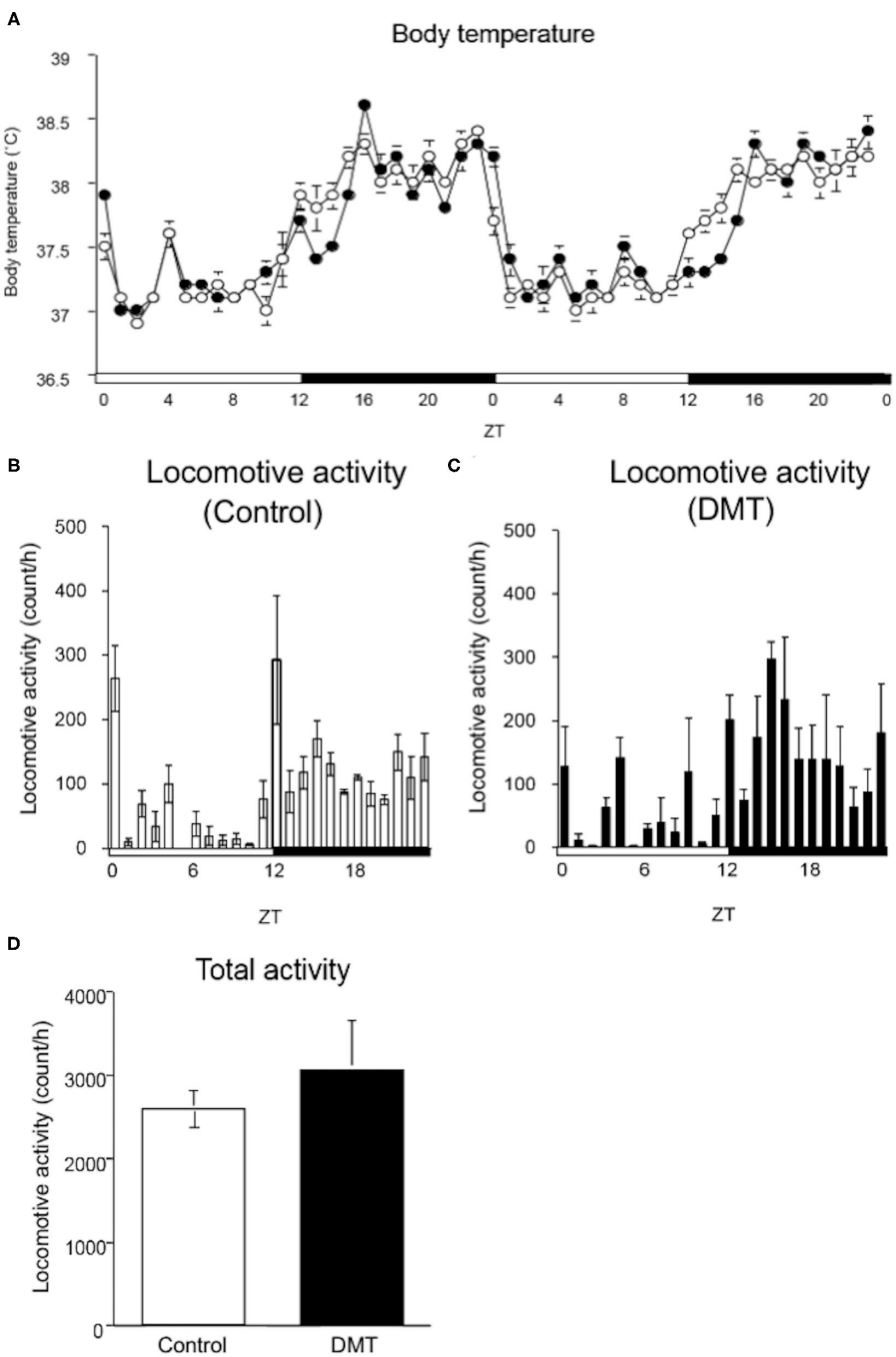
DMT delayed the surge in body temperature, although locomotive activity was not changed. **(A)** The body temperature was monitored during the experimental period (16–17 days). A temperature data logger was implanted into the intraperitoneal cavity of rats, which was removed after the experimental period. The open circles indicate the control group (°), and the solid circles indicate the DMT group (•). The body temperature was analyzed at a 60-min interval for 2 days. The body temperature value is presented as mean ± SEM (*n* = 5 in control group, *n* = 6 in DMT group). The pattern of locomotive activity for 24 h in the **(B)** control and **(C)** DMT groups was monitored. **(D)** The total locomotive activity of control and DMT rats was measured for 24 h during the experimental period. The total locomotive activity is expressed in terms of mean ± SEM (*n* = 3). The open and solid horizontal bars indicate the light (ZT 0-12) and dark (ZT 12-24) periods, respectively. The open and solid vertical bars indicate the control and DMT, respectively. The results of the rhythmicity analysis for body temperature and locomotive activity using JTK_CYCLE are shown in Supplementary Table 2.

The results were expressed as mean ± standard error of the mean (SEM). Statistical differences between the control and DMT groups were analyzed using the Student's *t*-test (Figures 1, 2D).

## Results

### DMT Rats Fed a High-Cholesterol Diet Showed No Change in Body Weight

We established a DMT protocol for one kind of breakfast skipping model in which rats consumed a diet after a 4 h delay during the active phase (29). The control rats ate 6.2 g (35.2%), 4.4 g (25.0%), and 7.0 g (39.8%) at ZT 12-16, ZT 16-20, and ZT 20-24, respectively. The DMT rats ate 8.0 g (45.7%), 6.7 g (38.3%), and 2.8 g (16.0%) at ZT 16-20, ZT 20-24, and ZT 24-4, respectively (Figure 1A). During ZT 16-20, amount of the food intake of the DMT rats was higher than that of the control rats (*p* < 0.001), whereas it did not change during ZT 20-0 (Figure 1A). The total food intake during the experimental period in the control and DMT groups was almost equal (*p* = 0.11) (Figure 1A, inset). Body weight gain was similar in both groups (Figure 1B).

### DMT Led to Increased Hepatic Lipids and Adipose Tissue Weight

There was no significant difference in the liver weight between the two groups (Figure 1C), but the epididymal adipose tissue weight increased in the DMT group (Figure 1D). It is known that the consumption of a high-cholesterol diet with cholate induces fatty liver in rats (35). The hepatic total lipid, triglycerides, and cholesterol levels increased in response to DMT (Figures 1E–G). However, the hepatic phospholipid levels did not change (Figure 1H).

### DMT Delayed the Surge in Body Temperature, But Not the Drop

The circadian change in body temperature is controlled by the hypothalamic thermoregulatory center, and the rhythm of the locomotive activity is regulated by the hypothalamus (39, 40). We monitored the daily rhythm of the body temperature and locomotive activity in the DMT rats (Figure 2). As rats are nocturnal, their body temperature is elevated when the light was turned off (ZT 12) and decreased when the light was turned on (ZT 24). However, the body temperature of the DMT rats increased gradually at ZT 12 and sharply at ZT 16 when they started feeding (Figure 2A). Furthermore, the diurnal rhythm of the body temperature was delayed by 0.5 h in response to DMT (Figure 2A and Supplementary Table 2). The diurnal variation in locomotive activity was also similar between the two groups (Figures 2B,C and Supplementary Table 2). There was no difference in the total locomotive activity between the groups (Figure 2D). These data suggest that locomotive activity is primary controlled by light conditions. We speculated that as a stimulus to body temperature, the timing of feeding was equally important as light.

### DMT Delayed the Diurnal Oscillation of Serum Nefa and Insulin Levels

**Figure 3:**
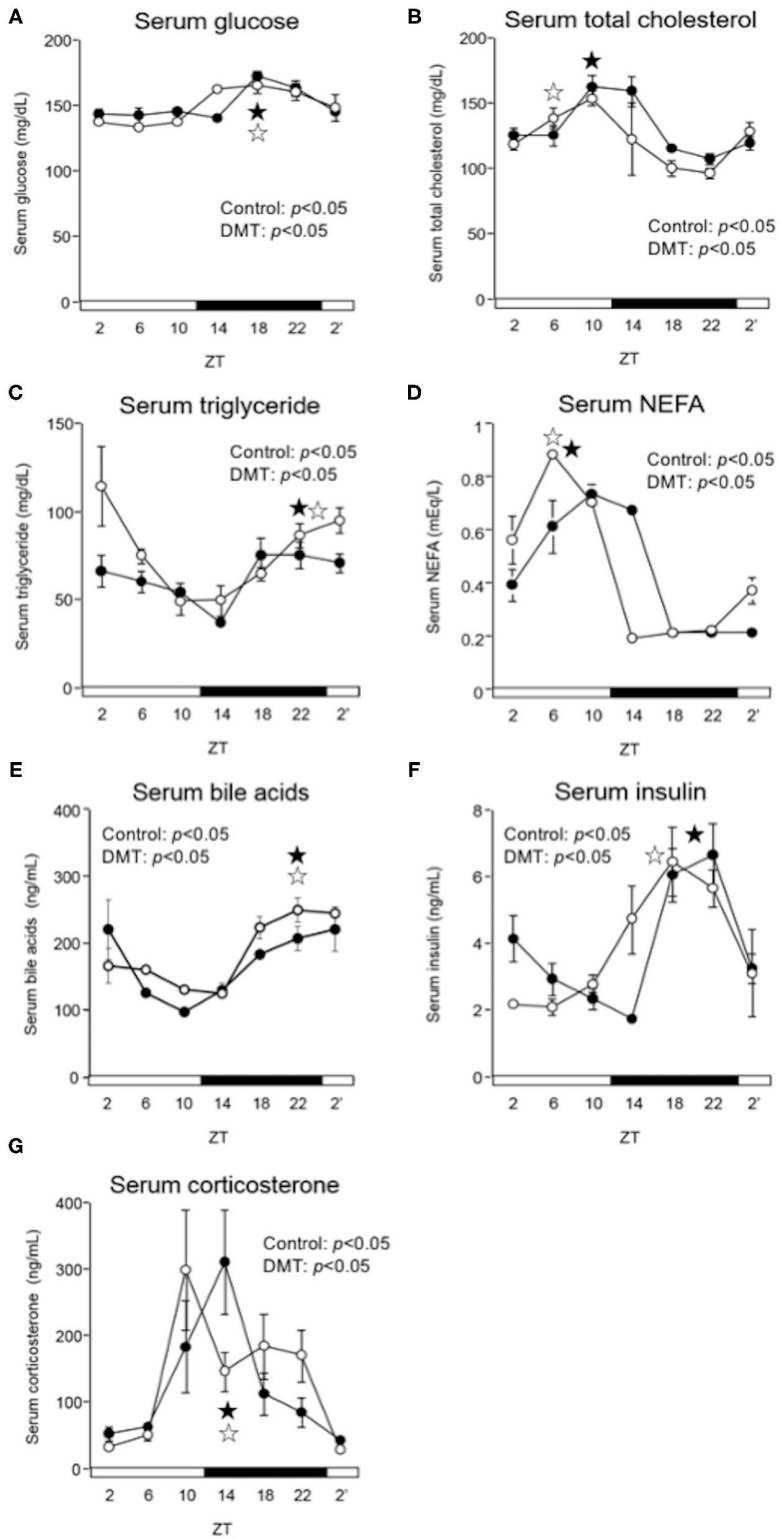
DMT delayed the peak of serum NEFA, and insulin in serum. We measured the serum concentrations of **(A)** glucose, **(B)** total cholesterol, **(C)** triglyceride, **(D)** NEFA, **(E)** bile acids, **(F)** insulin, and **(G)** corticosterone. Rats serum was harvested for seven times at 4 h interval on day 16–17. The open and solid horizontal bars indicate the light (ZT 0-12) and dark (ZT 12-24) periods, respectively. The values of serum parameters are presented as mean ± SEM (*n* = 3–4). The open circles indicate the control group (°), and the solid circles indicate the DMT group (•). The results of rhythmicity analysis for serum parameters using JTK_CYCLE are shown in Supplementary Table 2. The open star (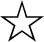) and the solid star (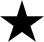) represent peak time points of circadian oscillation in the control and DMT groups, as analyzed using JTK_CYCLE (Supplementary Table 2), respectively.

In the DMT group, the diurnal rhythm of serum glucose and bile acids did not change (Figures 3A,E and Supplementary Table 2), whereas that of serum NEFA was delayed by 2 h (Figure 3D and Supplementary Table 2). Moreover, the diurnal rhythm of serum total cholesterol and insulin was delayed by 4 h in the DMT rats (Figures 3B,F and Supplementary Table 2). In contrast, the diurnal rhythm of serum triglyceride was advanced by 2 h in the DMT rats (Figure 3C and Supplementary Table 2). The level of serum corticosterone, secreted by the adrenal glands, is controlled by the adrenocorticotropic hormone secreted from the pituitary gland (41). Reportedly, corticosterone level is dependent on the timing of eating (42). However, in this study, the diurnal rhythm of serum corticosterone appeared to be delayed in DMT rats, but the change was not significant (Figure 3G and Supplementary Table 2).

### DMT Marginally Altered the Circadian Rhythm of the Hepatic Clock Genes

**Figure 4:**
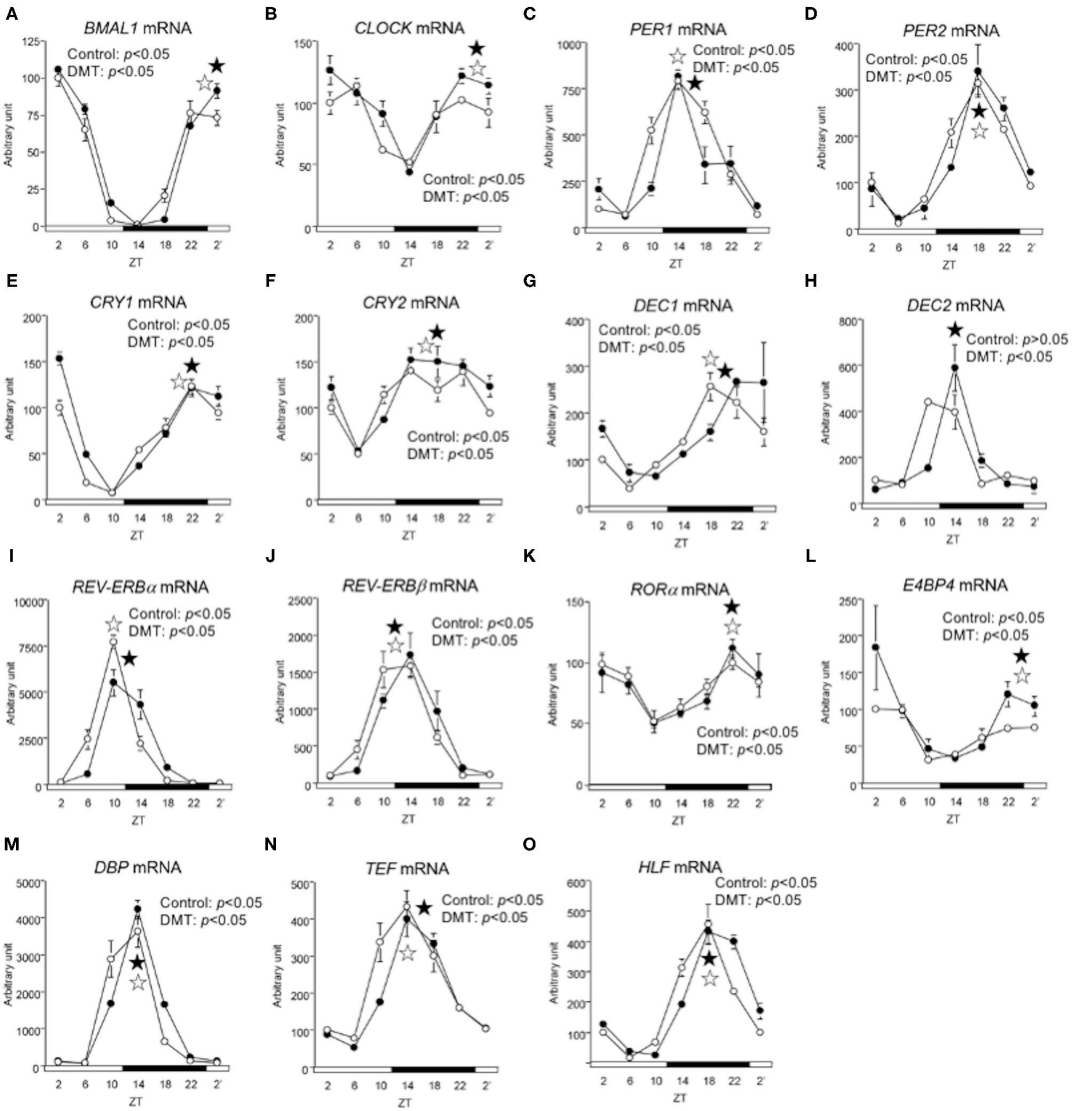
The circadian oscillations of hepatic circadian clock genes were consistent in response to DMT. We profiled the circadian oscillations of hepatic clock genes. We measured the circadian oscillations of **(A)** *BMAL1*, **(B)** *CLOCK*, **(C)** *PE*R1, **(D)** *PER2*, **(E)** *CRY1*, **(F)** *CRY2*, **(G)** *DEC1*, **(H)** *DEC2*, **(I)** *REV-ERBa*, **(J)** *REV-ERB*β **(K)** *ROR*α, **(L)** *E4BP4*, **(M)** *DBP*, **(N)** *TEF*, and **(O)** *HLF* genes with respect to the hepatic circadian clock. The mRNA levels were analyzed using real-time PCR, and the levels were normalized to the *Apo E* mRNA levels. The open and solid horizontal bars indicate the light (ZT 0-12) and dark (ZT 12-24) periods, respectively. The value of mRNA levels are expressed in terms of means ± SEM (*n* = 3–4). The open circles indicate the control group (°), and the solid circles indicate the DMT group (•). The results of rhythmicity analysis for hepatic circadian clock genes using JTK_CYCLE are shown in Supplementary Table 3. The open star (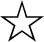) and the solid star (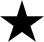) represent peak time points of circadian oscillation in the control and DMT groups, as analyzed using JTK_CYCLE (Supplementary Table 3), respectively. List of gene names we used in the present study is provided in Table 1.

We demonstrated that DMT rats fed a high-fat diet clearly showed a delay in clock gene expression (29). Therefore, we expected that the clock gene expression would be delayed in response to DMT, even in rats fed a high-cholesterol diet. However, the expression of *CLOCK, PER2*, and *DEC2* did not change (Figures 4B,D,H and Supplementary Table 3). In addition, the oscillations of *REV-ERB*β, *ROR*α, *E4BP4, DBP*, and *HLF* expression were also unaltered (Figures 4J–N and Supplementary Table 3). The expression of clock genes including *CLOCK, PER2, DEC2, REV-ERB*β, *ROR*α, *E4BP4, DBP*, and *HLF*, did not change. However, the other half of the clock genes including *BMAL1, PER1, CRY1, CRY2, DEC1, REV-ERB*α, and *TEF*, were delayed by 2 h in response to DMT (Figures 4A,C,E–G,I,O and Supplementary Table 3). These results showed that the circadian rhythms of hepatic clock gene expression were relatively consistent in DMT rats fed a high-cholesterol diet.

### DMT Delayed the Circadian Oscillation of Lipid Metabolism-Related Genes

**Figure 5:**
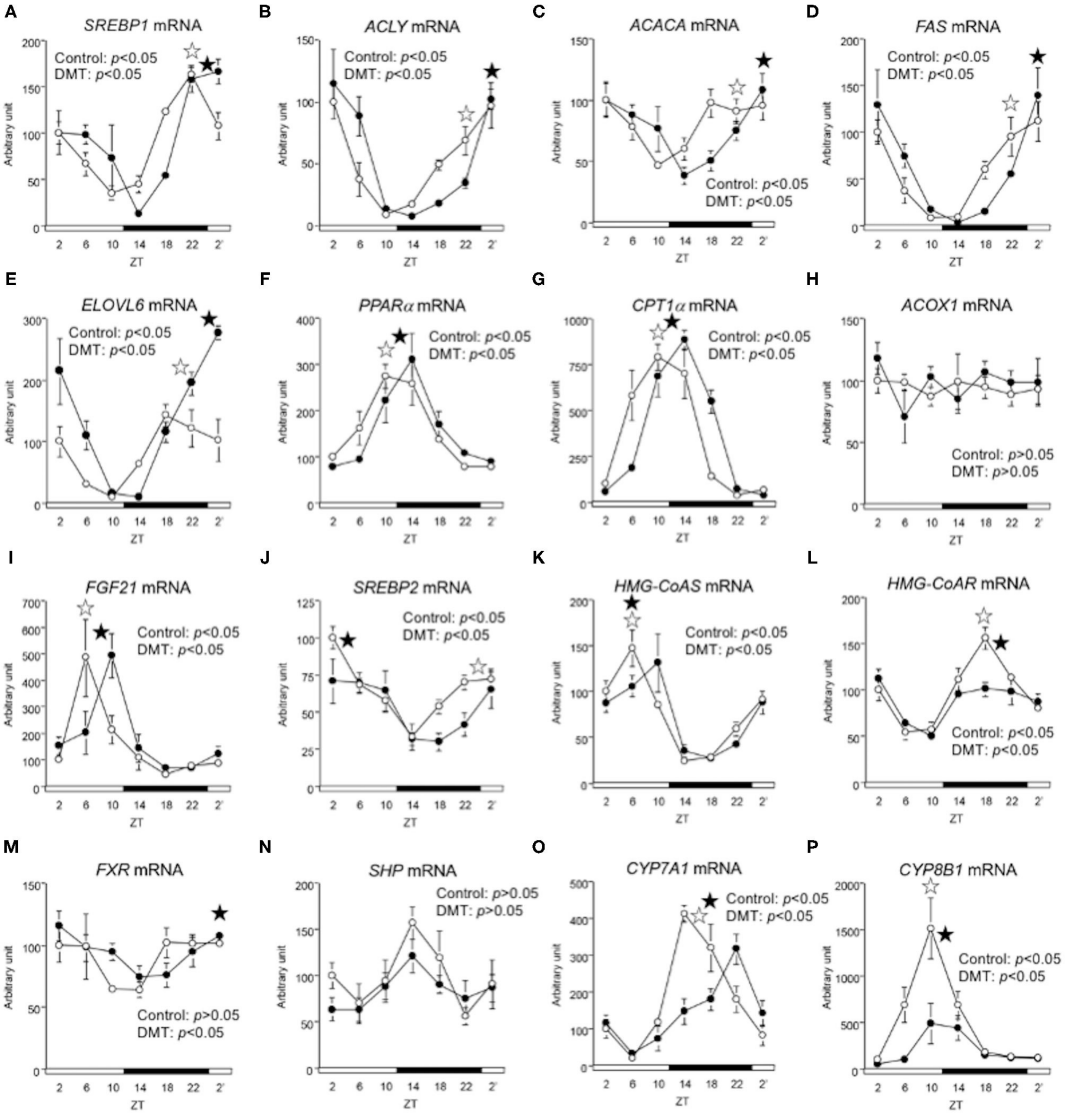
The circadian oscillations of hepatic lipid metabolism-related genes were delayed by DMT. We profiled the circadian oscillations of hepatic lipid metabolism-related genes. We measured the circadian expressions of **(A)** *SREBP1*, **(B)** *ACLY*, **(C)** *ACACA*, **(D)** *FAS*, and **(E)** *ELOVL6*, which are fatty acid synthesis-related genes, and of **(F)** *PPAR*α, **(G)** *CPT1*α, **(H)** *ACOX1*, and **(I)** *FGF21*, which are fatty acid oxidation-related genes. Moreover, the circadian expression of **(J)** *SREBP2*, **(K)** *HMG-CoAS*, and **(L)** *HMG-CoAR*, which are cholesterol synthesis-related genes, and of **(M)** *FXR*, **(N)** *SHP*, **(O)** *CYP7A1*, and **(P)** *CYP8B1*, genes related bile acid synthesis-related genes was also measured. The mRNA levels were analyzed using a real-time PCR, and the levels were normalized to the *Apo E* mRNA levels. The open and solid horizontal bars indicate the light (ZT 0-12) and dark (ZT 12-24) periods, respectively. The value of mRNA levels are expressed in terms of means ± SEM (*n* = 3–4). The open circles indicate the control group (°), and the solid circles indicate DMT group (•). The results of the rhythmicity analysis for hepatic lipid metabolism-related genes using JTK_CYCLE are shown in Supplementary Table 4. The open star (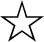) and the solid star (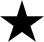) represent peak time points of circadian oscillation in the control and DMT groups, as analyzed using JTK_CYCLE (Supplementary Table 4), respectively. List of gene names we used in the present study was shown in Table 1.

We measured the expression of genes related to lipid metabolism. The oscillation of lipogenic genes such as *ACLY, ACACA, FAS, ELOVL6*, and *SREBP1* was delayed by 4 h in the DMT group (Figures 5A–E and Supplementary Table 4). The expression of *SREBP2* gene, which related to cholesterol synthesis, and *FXR* gene, which related to bile acids synthesis, was delayed by 4 h in the DMT group (Figures 5J,M). The expression of fatty acid oxidation-related genes, such as *PPAR*α, *CPT-1*α, and *ACOX1*, was delayed by 2 h in the DMT group (Figures 5F–H and Supplementary Table 4). Moreover, the oscillation of *FGF21*, controlled by *PPAR*α, was also delayed by 2 h in the DMT group (Figure 5I). The oscillation of *HMG-CoAS* remained unaltered (Figure 5K and Supplementary Table 4), however, *HMG-CoAR*, which related to cholesterol synthesis, and *SHP, CYP7A1*, and *CYP8B1*, which are related to bile acids synthesis was delayed by 2 h in the DMT group (Figures 5L,N–P and Supplementary Table 4). These results showed that the circadian oscillation of hepatic lipid metabolism was delayed by 2–4 h in response to DMT.

### DMT Slightly Affected Circadian Oscillations of Clock Genes in Epididymal Adipose Tissue

In the present study, DMT with a high-cholesterol diet increased epididymal adipose tissue weight (Figure 1D). We evaluated the circadian oscillation of clock genes in the epididymal adipose tissue. The peaks of *BMAL1, PER2*, and *CRY1* expression were not changed in response to DMT (Supplementary Figures 1a,d,e and Supplementary Table 5), whereas the peaks of *PER1, E4BP4*, and *DBP* expression were delayed by 2 h in response to DMT (Supplementary Figures 1c,g,h and Supplementary Table 5). Meanwhile, the peak of *CLOCK* expression was advanced by 2 h in response to DMT (Supplementary Figure 1b and Supplementary Table 5). The oscillation of *HMG-CoAS* remained unaltered (Supplementary Figure 1f and Supplementary Table 5). These results showed that the circadian rhythm of clock gene expression in epididymal adipose tissue was slightly altered by DMT.

## Discussion

Daily feeding rhythms are recognized as important factors for maintaining normal metabolism in humans (43). It is reported that skipping the first meal during an active period is an environmental risk factor that leads to a metabolic syndrome as it induces an increase in lipogenesis (44, 45). Currently, breakfast skipping is a major irregular feeding behavior prevalent in the society (46). Multiple studies have investigated the relationship between breakfast skipping and metabolic disorders in humans (23–27). However, it is difficult to identify the molecular mechanism by which breakfast skipping increases the risk of metabolic diseases in humans. It is known that the circadian rhythms of hepatic clock gene expression are altered by feeding behavior (10, 11, 47). It has also been reported that feeding time affects the circadian rhythm of hepatic lipid metabolism (13, 48). We found that irregular feeding induced hypercholesterolemia in rats (10). These findings indicate that feeding time and lipid metabolism are closely linked. The issue of breakfast skipping has not been considered to be caused by an abnormal circadian rhythm (49–52). We hypothesized that breakfast skipping induces metabolic disorders by inducing abnormalities in the hepatic circadian clock abnormalities. We recently demonstrated that DMT, a one kind of breakfast skipping model, led to increased body weight, delayed oscillations of hepatic clock genes and lipid metabolism-related genes in rats fed a high-fat diet (29). In the present study, we adopted the same protocol for rats fed a high-cholesterol diet. A high-cholesterol diet induces fatty liver and hypercholesterolemia, which are well-known risk factors for NAFLD and atherosclerosis, respectively. We hypothesized that DMT promoted fatty liver and hypercholesterolemia. In the present study, we found that DMT increased the hepatic lipid and adipose tissue weight without promoting hypercholesterolemia, even though the rats consumed almost the same quantity of food. The differences in the results between DMT with a high-fat diet (29) and a high-cholesterol diet are discussed below.

Oscillation in body temperature and locomotor activity are thought to be controlled by pacemakers present within the hypothalamus (53). With respect to the DMT group, the surge in the body temperature was delayed, although the drop in body temperature was not (Figure 2A). Therefore, surge in body temperature was regulated not only by light but also by feeding, and the drop in body temperature was strongly regulated by light (Figure 2A) (29). To compare the energy expenditure between the two groups, the area indicating the body temperature during ZT 12-16 and ZT 0-4 between groups was calculated (Figure 2A). The area corresponding to ZT 12-16 was ~2.1-fold larger than that corresponding to ZT 0-4. These data indicated that the energy expenditure was reduced owing to a 4 h delay in the surge of body temperature, which increased the liver lipid and adipose tissue weight in the DMT rats. On the other hand, the diurnal pattern of locomotive activity in the DMT group was similar to that in the control group (Figures 2B–D and Supplementary Table 2). From these results suggested that locomotive activity was strongly regulated by light, but not feeding (Figures 2B,C).

In the present study, the oscillation in the expression of *de novo* fatty acid synthesis genes were clearly delayed by 4 h in response to DMT (Figure 5 and Supplementary Table 4). These delays in lipid metabolism would be related to the lipid accumulation in the liver (Figures 2E–G, 5 and Supplementary Table 4). However, the circadian rhythms of the hepatic clock gene expression were relatively consistent in DMT rats fed a high-cholesterol diet (Figure 4). The mammalian circadian clock is generally composed of a hierarchical circadian clock system. Circadian oscillation of metabolic genes is regulated by clock genes (4, 7, 9). Indeed, in DMT rats fed a high-fat diet, the oscillation of clock genes and lipid metabolism-related gene expression was delayed by 4 h (29). These data indicated that in DMT rats fed a high-cholesterol diet, the hepatic hierarchical circadian clock system was disintegrated. In both DMT studies, insulin level was delayed by 4 h (29) (Figure 3F and Supplementary Table 2). Therefore, the delay in insulin level induced the 4 h delay in the expression of lipid metabolism-related genes (Figures 3F, 5). Although DMT with a high-fat diet led to a shift in the liver core clock rhythm (29), the core circadian clock in the liver was relatively consistent when DMT rats were fed a high-cholesterol diet (Figure 4). We hypothesized that simultaneous shifts in factors other than insulin, such as, NEFA and bile acids, would be required to shift the liver core clock oscillations (29). The peaks of serum NEFA and insulin levels were delayed (Figures 3D,F and Supplementary Table 2). However, the levels of bile acids were shifted only by DMT with a high-fat diet (29), but not by DMT with a high-cholesterol diet (Figure 3E and Supplementary Table 2). Bile acids have been reported to change the oscillation of core clock gene expression (54). This indicated that a simultaneous shift in bile acids and insulin would be required to shift the liver core clock.

In the present study, DMT in rats fed a high-cholesterol diet induced hepatic lipids accumulation and increased adipose tissue weight even at similar food intake and body weight. The surge in body temperature was delayed by 4 h in the DMT group, suggesting that energy expenditure was decreased by DMT. In addition, the circadian rhythm of hepatic lipid metabolism-related genes was clearly 2–4 h by DMT, although the circadian rhythm of hepatic clock genes was 2 h delayed or not. These results indicated that DMT with a high-cholesterol diet induced the disintegration of the hepatic hierarchical circadian clock system, and the circadian rhythm of *de novo* fatty acid metabolism was controlled by the timing of, eating but not by directly *via* the controlling of clock genes. Taken together, the findings indicate that breakfast, which is the first meal during an active period, is one of important cue for lipid metabolism homeostasis.

## Data Availability Statement

The raw data supporting the conclusions of this article will be made available by the authors, without undue reservation.

## Ethics Statement

The animal study was reviewed and approved by The Animal Care Committee of Nagoya University, Nagoya University.

## Author Contributions

DK and FH conceptualized this study, curated data, wrote the original draft, and reviewed and edited the final draft. SS, TL, SI, MU, and SM curated data. HO conceptualized this study, administered project, designed methodology, reviewed and edited the final draft, and acquired funding for the studies. All authors contributed to the article and approved the submitted version.

## Conflict of Interest

The authors declare that the research was conducted in the absence of any commercial or financial relationships that could be construed as a potential conflict of interest.
