# Supplementary material for: Delayed Meal Timing, a Breakfast Skipping Model, Increased Hepatic Lipid Accumulation and Adipose Tissue Weight by Disintegrating Circadian Oscillation in Rats Fed a High-Cholesterol Diet
Source: Front Nutr. 2021 Jul 1;8:681436. doi: 10.3389/fnut.2021.681436 (PMC8280346; doi:10.3389/fnut.2021.681436)
Supplement: Supplementary file 1 [file Image_1.pdf]

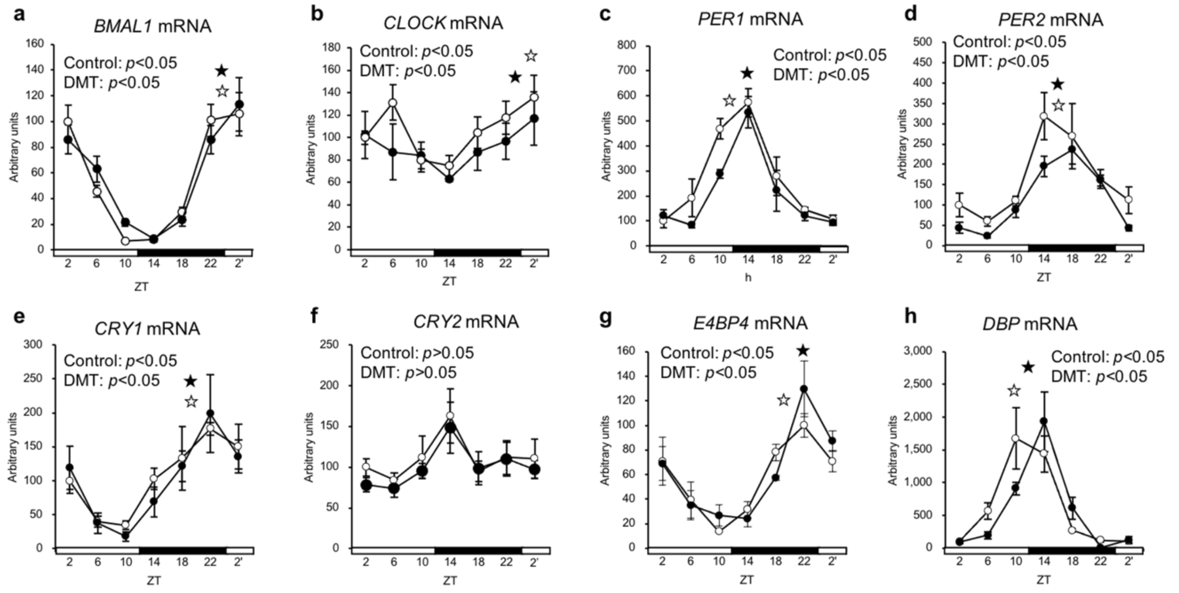

Supplementary Figure 1. DMT slightly affected circadian oscillations of clock genes in epididymal adipose tissue. We profiled circadian oscillations of clock genes in epididymal adipose tissue. We measured circadian oscillations of (a) *BMAL1*, (b) *CLOCK*, (c) *PE*R1, (d) *PER2*, (e) *CRY1*, (f) *CRY2*, (g) *E4BP4*, (h) *DBP* genes related circadian clock in epididymal adipose tissue. The mRNA levels were analyzed using a real-time PCR, and these were normalized by *18S* rRNA level. The open and solid horizontal bars indicate the light (ZT 0-12) and dark (ZT 12-24), respectively. The value of mRNA levels are means ± SEM (n=3-4). The open circles indicate control group (○) and the solid circles indicate DMT group (●). The results of rhythmicity analysis for hepatic circadian clock genes using JTK_CYCLE are shown in Supplementary Table 5. The open star (☆) and the solid star (★) represent peak time points of circadian oscillation in the control and DMT groups, as analyzed using JTK_CYCLE (Supplementary Table 5), respectively. List of gene names we used in the present study is provided in Table 1.
