# Supplementary material for: Delayed Meal Timing, a Breakfast Skipping Model, Increased Hepatic Lipid Accumulation and Adipose Tissue Weight by Disintegrating Circadian Oscillation in Rats Fed a High-Cholesterol Diet
Source: Front Nutr. 2021 Jul 1;8:681436. doi: 10.3389/fnut.2021.681436 (PMC8280346; doi:10.3389/fnut.2021.681436)
Supplement: Supplementary file 2 [file Table_1.docx]

**Supplementary Table 1.** Primer sequences for quantitative real-time PCR.

| **Gene** | **Accession No.** | **Forward primer ( 5'→3' )** | **Reverse Primer ( 5'→3' )** |
| --- | --- | --- | --- |
| *18S* | NR_003286 | CGCCGCTAGAGGTGAAATTC | TTGGCAAATGCTTTCGCTC |
| *ACACA* | NM_022193.1 | GCCAGCAGAATTTGTTACTC | ATAAGACCACCGCCGGATAG |
| *ACLY* | NM_016987.2 | TGGACTTTGACTACGTGTGC | AAGACAGGGATCAGGATTTC |
| *ACOX1* | NM_017340.2 | TGATGAAATACGCCCAGGTG | CCCATACGTCAGCTTGTTAC |
| *APO E* | NM_001270681.1 | TGAACCGCTTCTGGGATTAC | TGGGTGACTTGGGAGCTCTG |
| *BMAL1* | NM_024362.2 | TTTGTAGATCAGAGGGCGAC | GTACCTAGAAGTTCCTGTGG |
| *CLOCK* | NM_021856.2 | AGTTAGGGCTGAAAGACGGC | TTCCAGTGCTTCCTTGAGAC |
| *CPT1α* | NM_031559.2 | GGGATTAAGGTTCTGCTATG | AAGATGTGTGAGGAAGGTGG |
| *CRY1* | NM_198750.2 | CACTATGCTCACGGAGACAG | CCCATGGAGCTTCTTCTTTG |
| *CRY2* | NM_133405.2 | CTGGATAAGCACTTGGAACG | ACAGGCGGTAGTAGAAGAGG |
| *CYP7A1* | NM_012942.2 | TGTGTGAGGGACCAGGTCTCT | AGCTCCAAAAGGTTGGAGGA |
| *CYP8B1* | NM_031241.1 | GCCTCTTCCACTTCTGCTAC | ACTTCCTGAACAGCTCATCG |
| *DBP* | NM_012543.3 | CTCTAGGGACACACCCAGTCCT | AGGCTTCAATTCCTCCTCTGAGA |
| *DEC1* | NM_053328.1 | GAAACCATTGGACTCAGCTC | TTTCCCAGGGCCTTCTGATC |
| *DEC2* | XM_002729454.6 | TAACAGCCTTAACGGAGCAG | CAAGACTTCTTTGGCGCAGG |
| *E4BP4* | NM_053727.2 | ACTGGCATCACAAAGAACTG | ACTGGCATCACAAAGAACTG |
| *ELOVL6* | NM_134383.2 | ACCCGAACTAGGTGATACG | CCCAGCTACCATGTCTTTG |
| *FAS* | NM_017332.1 | CCAAGCAGGCACACACAATG | GATACCTCCGTCGACAATAG |
| *FGF21* | NM_130752.1 | TCTACACAGATGACGACCAG | CACCCAGGATTTGAATGACC |
| *FXR* | NM_021745.1 | AGCCACAGATCTCCTCCTCG | ACAGGCATCTCGGATACCTCA |
| *HLF* | NM_001100764.1 | CGCTTTGCCTTCTGCTCATC | CCTGTGTAGGATGCTCTTTC |
| *HMG-CoAR* | NM_013134.2 | TGCACAGACTCCTCAGACGTG | TTCGTCAAAACACCAGCTTCC |
| *HMG-CoAS* | NM_017268.1 | ACACACATCACTTAGCCAAC | CCACTCCTTCATCCAAACTG |
| *PER1* | NM_001034125.1 | ACCAGCTCAAGGCTTAGGAGCT | TGGGATTTGGAGAGACCACTTC |
| *PER2* | NM_031678.1 | CAACCTTTGTCTGCCATATGAGG | CGTTAGAAACACAAGCTCTTCCAC |
| *PPARα* | NM_013196.1 | TTCGGCTAAAGCTGGCGTAC | ACTGGCATTTGTTCCGGTTC |
| *REV-ERBα* | NM_001113422.1 | CCTTTGAGGTGCTGATGGTG | ACATGACTGTCTGGTCCTTC |
| *REV-ERBβ* | NM_147210.2 | TGAACTTCTCCAGTGCTTAC | ACTTGCTCATAGGACAAACC |
| *RORα* | XM_008766408.2 | ACGATGACCTCAGCACCTAC | CACATATGGGTTCGGGTTTG |
| *SHP* | NM_057133.1 | CAGCTTGGATTTCCTCGGTTT | GTCTGGAGGAATTCTGCCCTG |
| *SREBP1c* | NM_001276708.1 | GGAGCCATGGATTGCACATT | AGGAAGGCTTCCAGAGAGGA |
| *SREBP2* | NM_001033694.1 | ACGGTGTGATTGTCTTGAGC | TTTGGCGAGGTCTAGGTCTG |
| *TEF* | NM_019194.2 | CCCTTGAGTATCAGCAGGTC | CAGTGTAATGTCTCCAGAGC |
