# Supplementary material for: Delayed Meal Timing, a Breakfast Skipping Model, Increased Hepatic Lipid Accumulation and Adipose Tissue Weight by Disintegrating Circadian Oscillation in Rats Fed a High-Cholesterol Diet
Source: Front Nutr. 2021 Jul 1;8:681436. doi: 10.3389/fnut.2021.681436 (PMC8280346; doi:10.3389/fnut.2021.681436)
Supplement: Supplementary file 3 [file Table_2.docx]

**Supplementary Table 2.** JTK_CYCLE analysis of circadian oscillations in body temperature and serum parameters by DMT (Related to Fig.2a, b, c and Fig.3).

|  |  | Control | | | | DMT | | |
| --- | --- | --- | --- | --- | --- | --- | --- | --- |
|  |  | *p*-value | Peak time (ZT) | Amplitude |  | *p*-value | Peak time (ZT) | Amplitude |
| Body temperature | | 0.00000 | 18.5 | 0.61283 |  | 0.00000 | 19 | 0.54408 |
| Locomotive activity | | 0.17800 | 18 | 58.69000 |  | 0.19100 | 17 | 58.69000 |
| Serum total cholesterol | | 0.00015 | 6 | 26.26196 |  | 0.00021 | 10 | 22.12516 |
| Serum corticosterone | | 0.00024 | 14 | 85.83236 |  | 0.00004 | 14 | 63.50133 |
| Serum glucose | | 0.00027 | 18 | 18.56943 |  | 0.00607 | 18 | 11.46195 |
| Serum insulin | | 0.00002 | 16 | 2.25291 |  | 0.00007 | 20 | 2.28878 |
| Serum NEFA | | 0.00000 | 6 | 0.37684 |  | 0.00006 | 8 | 0.30349 |
| Serum triglyceride | | 0.00000 | 0 | 21.15671 |  | 0.02631 | 22 | 15.70593 |
| Serum bile acids | | 0.00001 | 22 | 28.28360 |  | 0.00042 | 22 | 29.14134 |
