# Supplementary material for: Delayed Meal Timing, a Breakfast Skipping Model, Increased Hepatic Lipid Accumulation and Adipose Tissue Weight by Disintegrating Circadian Oscillation in Rats Fed a High-Cholesterol Diet
Source: Front Nutr. 2021 Jul 1;8:681436. doi: 10.3389/fnut.2021.681436 (PMC8280346; doi:10.3389/fnut.2021.681436)
Supplement: Supplementary file 4 [file Table_3.docx]

**Supplementary Table 3.** JTK_CYCLE analysis of circadian oscillations in hepatic clock genes by DMT (Related to Fig. 4).

| Hepatic  clock genes | Control | | | | DMT | | |
| --- | --- | --- | --- | --- | --- | --- | --- |
|  | *p*-value | Peak time (ZT) | Amplitude |  | *p*-value | Peak time (ZT) | Amplitude |
| *BMAL1* | 0.00000 | 0 | 50.68525 |  | 0.00000 | 2 | 52.853 |
| *CLOCK* | 0.00263 | 0 | 26.11993 |  | 0.00009 | 0 | 31.521 |
| *CRY1* | 0.00000 | 20 | 52.86544 |  | 0.00000 | 22 | 57.875 |
| *CRY2* | 0.00010 | 16 | 31.93166 |  | 0.00002 | 18 | 44.974 |
| *DBP* | 0.00001 | 14 | 1412.43307 |  | 0.00000 | 14 | 1249.212 |
| *DEC1* | 0.00000 | 18 | 86.41853 |  | 0.00001 | 20 | 79.391 |
| *DEC2* | 0.06592 | 12 | 144.43291 |  | 0.00000 | 14 | 96.625 |
| *E4BP4* | 0.00037 | 0 | 34.72907 |  | 0.00000 | 0 | 54.884 |
| *HLF* | 0.00000 | 18 | 170.91639 |  | 0.00000 | 18 | 207.440 |
| *PER1* | 0.00000 | 14 | 341.43152 |  | 0.00001 | 16 | 226.580 |
| *PER2* | 0.00000 | 18 | 129.07988 |  | 0.00000 | 18 | 136.816 |
| *REV-ERBα* | 0.00000 | 10 | 1882.08591 |  | 0.00000 | 12 | 2485.845 |
| *REV-ERBβ* | 0.00000 | 12 | 802.22105 |  | 0.00000 | 12 | 699.020 |
| *RORα * | 0.00012 | 22 | 18.54567 |  | 0.00021 | 22 | 20.000 |
| *TEF* | 0.00000 | 14 | 176.90906 |  | 0.00000 | 16 | 137.556 |
