# Supplementary material for: Delayed Meal Timing, a Breakfast Skipping Model, Increased Hepatic Lipid Accumulation and Adipose Tissue Weight by Disintegrating Circadian Oscillation in Rats Fed a High-Cholesterol Diet
Source: Front Nutr. 2021 Jul 1;8:681436. doi: 10.3389/fnut.2021.681436 (PMC8280346; doi:10.3389/fnut.2021.681436)
Supplement: Supplementary file 5 [file Table_4.docx]

**Supplementary Table 4.** JTK_CYCLE analysis of circadian oscillations in hepatic lipid metabolism related genes by DMT (Related to Fig.

5).

| Lipid metabolism-related genes | Control | | | | DMT | | |
| --- | --- | --- | --- | --- | --- | --- | --- |
|  | *p*-value | Peak time (ZT) | Amplitude |  | *p*-value | Peak time (ZT) | Amplitude |
| *ACACA* | 0.00628 | 22 | 25.85621 |  | 0.00003 | 2 | 24.93152 |
| *ACLY* | 0.00000 | 22 | 35.96006 |  | 0.00000 | 2 | 30.74938 |
| *ACOX1* | 1.00000 | 0 | 2.03799 |  | 1.00000 | 22 | 12.75358 |
| *CPT1α* | 0.00000 | 10 | 426.36197 |  | 0.00000 | 12 | 421.90037 |
| *CYP7A1* | 0.00000 | 16 | 167.29111 |  | 0.00014 | 18 | 90.50591 |
| *CYP8B1* | 0.00000 | 10 | 492.68428 |  | 0.00031 | 12 | 119.28745 |
| *ELOVL6* | 0.00001 | 20 | 61.24047 |  | 0.00000 | 0 | 91.44963 |
| *FAS* | 0.00000 | 22 | 46.60480 |  | 0.00000 | 2 | 37.15415 |
| *FGF21* | 0.00001 | 6 | 81.80201 |  | 0.00026 | 8 | 97.14146 |
| *FXR* | 0.05791 | 22 | 20.32757 |  | 0.00560 | 2 | 15.66689 |
| *HMG-CoAR* | 0.00000 | 18 | 39.84434 |  | 0.00144 | 20 | 23.87533 |
| *HMG-CoAS* | 0.00006 | 4 | 41.90268 |  | 0.00014 | 4 | 47.21278 |
| *PPARα* | 0.00000 | 10 | 88.09148 |  | 0.00001 | 12 | 84.38261 |
| *SHP* | 0.41562 | 14 | 33.48899 |  | 0.18853 | 12 | 22.69219 |
| *SREBP1c* | 0.00002 | 22 | 47.88885 |  | 0.00000 | 0 | 60.06859 |
| *SREBP2* | 0.00001 | 0 | 19.89491 |  | 0.00560 | 4 | 25.64937 |
