# Supplementary material for: Delayed Meal Timing, a Breakfast Skipping Model, Increased Hepatic Lipid Accumulation and Adipose Tissue Weight by Disintegrating Circadian Oscillation in Rats Fed a High-Cholesterol Diet
Source: Front Nutr. 2021 Jul 1;8:681436. doi: 10.3389/fnut.2021.681436 (PMC8280346; doi:10.3389/fnut.2021.681436)
Supplement: Supplementary file 6 [file Table_5.docx]

**Supplementary Table 5.** JTK_CYCLE analysis of circadian oscillations in clock genes in epididymal adipose tissue by DMT (Related to Supplementary Figure 1).

| clock genes  in epididymal  adipose tissue | Control | | | | DMT | | |
| --- | --- | --- | --- | --- | --- | --- | --- |
|  | *p*-value | Peak time (ZT) | Amplitude |  | *p*-value | Peak time (ZT) | Amplitude |
| *BMAL1* | 0.00000 | 0 | 48.2137317 |  | 0.00000 | 0 | 41.6984046 |
| *CLOCK* | 0.03876 | 2 | 20.7986274 |  | 0.68884 | 0 | 7.05376422 |
| *CRY1* | 0.00008 | 20 | 53.7121224 |  | 0.00014 | 20 | 64.279776 |
| *CRY2* | 1.00000 | 18 | 14.9049699 |  | 0.49130 | 12 | 14.1044463 |
| *DBP* | 0.00000 | 10 | 573.819759 |  | 0.00000 | 12 | 622.32418 |
| *E4BP4* | 0.00000 | 20 | 58.8188574 |  | 0.00000 | 22 | 49.7783193 |
| *PER1* | 0.00000 | 12 | 219.080038 |  | 0.00014 | 14 | 143.700769 |
| *PER2* | 0.00008 | 16 | 90.2642501 |  | 0.00000 | 16 | 102.536062 |
